# Alcohol disorder amongst forcibly displaced persons in northern Uganda

**DOI:** 10.1016/j.addbeh.2011.03.006

**Published:** 2011-08

**Authors:** Bayard Roberts, Kaducu Felix Ocaka, John Browne, Thomas Oyok, Egbert Sondorp

**Affiliations:** a Faculty of Public Health and Policy, London School of Hygiene and Tropical Medicine, 15-17 Tavistock Place, London WC1H 9SH, United Kingdom; b Faculty of Medicine, Gulu University, PO Box 166, Gulu, Uganda; c Department of Epidemiology and Public Health, University College Cork, Ireland

**Keywords:** Alcohol, Uganda, War, Risk-factors

## Abstract

**Background:**

Alcohol use may be a coping mechanism for the stressors related to forced displacement. The aim of this study was to investigate levels and determinants of alcohol disorder amongst internally displaced persons (IDPs) in northern Uganda.

**Methods:**

A cross-sectional survey with 1206 adult IDPs was conducted in Gulu and Amuru districts. Alcohol disorder was measured using the AUDIT instrument. Multivariate logistic regression was used to explore demographic, socio-economic, displacement and trauma exposure determinants of alcohol disorder.

**Findings:**

The prevalence of probable alcohol disorder was 17% of all respondents, and 66% amongst those who drank alcohol once a month or more frequently. Factors associated with alcohol disorder included men compared to women, older age, and experiencing a higher number of traumatic events. These findings can help identify potentially vulnerable groups and target responses more effectively.

## Research highlights

► 80% of sample reported drinking once a month or less (63% men, 91% women). ► 17% of all sample categorised as having alcohol disorder (32% men, 7% women). ► 66% of those drinking >monthly categorised with alcohol disorder (76% men, 47% women). ► Men, older age, and exposure to traumatic events associated with harmful alcohol use.

## 1 Background

Concern has been raised about alcohol disorder amongst persons forcibly displaced from their home areas due to war and persecution (Johnson, 1996; UNHCR & WHO, 2008). These forcibly displaced persons include approximately 27 million internally displaced persons (IDPs) who have fled their homes but remain within the borders of their own country, and 16 million refugees who have crossed into another country. Forcibly displaced persons can be exposed to a high number of traumatic and violent events and may subsequently experience post-traumatic stress disorder (Porter & Haslam, 2005; Steel et al., 2009), both of which may be risk factors for alcohol disorder (Stewart, 1996).

Alcohol use may also be a coping mechanism for other commonly experienced stressors of forced displacement such as poor living conditions, impoverishment, unemployment, boredom, and the loss of self-esteem and cultural and social support high rates of depression, anxiety and generalised psychological distress have been recorded with forcibly displaced populations (de Jong, Komproe, & Van Ommeren, 2003), and there is evidence of their co-morbidity with alcohol disorder (Kessler et al., 1997; Sacco, Bucholz, & Spitznagel, 2009).

Despite these potential risk factors for alcohol disorder, a recent systematic review noted a weak evidence-base on determinants of alcohol use amongst forcibly displaced persons (Weaver & Roberts, 2010), and the evidence was limited to high- and high/middle-income countries whereas the majority of displaced persons live in low-income countries where patterns and determinants of alcohol use may differ.

The aim of this study was to investigate levels and determinants of alcohol disorder amongst IDPs in northern Uganda.

### 1.1 Internal displacement in northern Uganda

The study setting of northern Uganda has witnessed a war since 1986 between the Ugandan government and the Lord's Resistance Army (LRA) rebel movement. The civilian population suffered extreme violence, and up to 2 million people forcibly displaced into IDP camps (Human Rights Watch, 2005).

The camps were characterised by severe over-crowding, poor water and sanitation, impoverishment, food aid dependency, restricted movement beyond the camps, and violent attacks (Human Rights Watch, 2005; Roberts, Ocaka, Browne, Oyok, & Sondorp, 2008). In August 2006, a ceasefire was signed resulting in improved security and return of most IDPs to their homes from 2008 onwards.

There has been speculation about alcohol disorder amongst the conflict-affected population in northern Uganda (Gulu District Sub Working Group On Sexual & Gender Based Violence, 2005; Huber, 2010; International Medical Corps, 2011). However, no studies could be identified quantifying alcohol use amongst IDPs in northern Uganda.

## 2 Methods

The findings from this study are from a broader study which took place in 2006 in Gulu and Amuru districts of northern Uganda (for further details see (Roberts, Felix Ocaka, Browne, Oyok, & Sondorp, 2009; Roberts et al., 2008)). At the time of the study, the two districts contained an estimated 650,000 IDPs which was approximately 40% of all IDPs in Uganda. Up to 80% of the districts' population lived in IDP camps ranging in size from around 1000 to 60,000 people (WFP, 2006).

The study involved a cross-sectional survey using a face-to-face orally administered questionnaire delivered by trained lay people. The main outcome of interest for this paper is alcohol disorder which was measured by using the Alcohol Use Disorders Identification Test (AUDIT) (Babor, Biddle, Saunders, & Monteiro, 2001). AUDIT was developed for international use and provides an accurate measure of alcohol disorder risk across cultures, gender and age (Allen, Litten, Fertig, & Babor, 1997; Reinert & Allen, 2002; Saunders, Aasland, Babor, de la Fuente, & Grant, 1993). AUDIT consists of 10 items addressing the frequency, quantity, and effect of drinking with a recall period of the previous 1 year. Each item has a response scoring range from 0 to 4 and response scores are summed to produce a total score with higher scores indicating more likelihood of alcohol disorder. Alcohol included manufactured beer, homebrewed beer, and distilled alcohol beverages including waragi which is a strong locally distilled spirit, as these were the types of alcohol available in and around the IDP camps. ‘A drink’ was defined as 1 glass of waragi, 1 bottle or glass of local brew or 1 bottle of beer.

The questionnaire also included items on demographic, socio-economic and forced displacement characteristics (longevity, distance, frequency), and exposure to 16 traumatic events using the Harvard Trauma Questionnaire (Mollica, Massagli, & Silove, 2004). The questionnaire was developed in English and translated and delivered in Acholi, the main language of Gulu and Amuru districts.

The study used a cross-sectional survey design using multi-stage cluster sampling (for details on sampling see (Roberts et al., 2008)), and the sample population was adult (≥ 18 years old) male and female IDPs. IDPs were defined as people living in officially recognised IDP camps. Fifteen data collectors administered the questionnaire (8 men and 7 women) who were all from the Acholi region of northern Uganda and spoke fluent Acholi and English. The data collection took place between the 6 and 27 of November 2006.

Ethical approval for the study was provided by the Ugandan National Council for Science and Technology, Gulu University, and the London School of Hygiene and Tropical Medicine.

Data analysis included the frequency of alcohol use and the following typologies of alcohol use: current abstainer (never had a drink or had none in the past year); infrequent light drinker (drinking < weekly and always < 5 drinks per occasion); frequent light drinker (drinking ≥ weekly and < 5 drinks per occasion); and infrequent heavy drinker (drinking ≥ weekly and sometimes ≥ 5 drinks per occasion) (Obot & Room, 2005). Respondent AUDIT scores were also summarised into 4 categories of: having no alcohol problem (≤ 7); advice on alcohol use suggested (8–15); counselling suggested (16–19); and treatment suggested (≥ 20), based upon AUDIT guidelines (Babor et al., 2001).

Logistic regression analysis was used to investigate respondent characteristics associated with alcohol disorder, applying a cut-off score of ≥ 8 (Babor et al., 2001). Bivariate analysis was firstly conducted to explore associations with demographic, socio-economic and displacement-related characteristics. The characteristics which showed statistically significant (P < 0.05) associations were then included in a multivariate analysis to adjust for the influence of other significant characteristics using a stepwise approach. The analysis was adjusted for the clustered design.

## 3 Results

There were a total of 1206 respondents and the response rate was 94%. There were more women (60.0%) than men, and the mean age of respondents was 35 years. Ten percent of respondents had completed secondary education. Seventy percent of respondents had been displaced for more than 5 years. Over half (57.7%) of respondents had experienced ≥ 8 of the 16 trauma events during their lifetime.

The frequency and typology of drinking alcohol is given in Table 1. Eighty percent of respondents reported that they drank alcohol only once a month or less (63.5% of men, 91.0% of women). In terms of typology, three quarters of respondents were abstainers (57.5% men, 85.2% women). Less than 2% of respondents reported being heavy drinkers (3.6% of men, 0.1% of women).

The mean AUDIT score was 5.8 for all men and 1.3 for all women, and the results for the different AUDIT categories are given in Table 1. 32.4% of men and 7.1% of women were categorised as having alcohol disorder. More specifically, 15.8% of men and 5.1% of women required advice on alcohol consumption, 7.7% of men and 0.6% of women required counselling on alcohol consumption, and 8.9% of men and 1.4% of women required treatment for their alcohol consumption. The prevalence of alcohol disorder amongst the 312 respondents that drank alcohol once a month or more frequently was 66.3% (76.1% of men, 47.7% of women).

The multivariate analysis (Table 2) highlights the pervasive influence of gender on alcohol disorder, with men 7 times more likely than women to be above the threshold level for alcohol disorder (OR 7.21 [95% CI 4.79; 10.86]). Older age shows a strong association with possible alcohol disorder, with respondents aged ≥ 50 4 times more likely to be above the threshold than respondents aged < 30 (OR 4.14 [95% CI 2.62; 6.52]). Respondents who experienced ≥ 12 events were twice as likely to have scores indicative of alcohol disorder than those who had experienced ≤ 3 events (OR 2.11 [95% CI 1.02; 4.38]). Post traumatic stress disorder, depression, and characteristics of displacement showed no association with alcohol disorder.

## 4 Discussion

The proportion of the study population drinking alcohol is slightly lower than findings of stable populations in Uganda (Tumwesigye & Kasirye, 2005). In terms of alcohol disorder, a study in Kampala recorded 17% of respondents being above a threshold for alcohol disorder which corresponds closely with our study findings (Kullgren, Alibusa, & Birabwa-Oketcho, 2009). Alcohol disorder was more prevalent among men in our study which reflects findings from other studies on alcohol use amongst forcibly displaced populations (Weaver & Roberts, 2010), and also in general populations in other countries (including Uganda) (Obot & Room, 2005; Tumwesigye & Kasirye, 2005; WHO, 2004). Cumulative exposure to traumatic events was also associated with alcohol use and this reflects other studies of forcibly displaced populations (Weaver & Roberts, 2010).

Our study findings suggest that speculation of substantially higher alcohol use amongst IDPs in northern Uganda than the rest of the Ugandan population may be unwarranted (although there are a number of study limitations discussed below). However, it appears to be the case that people in the study population that do drink alcohol are highly likely to have an alcohol disorder and would benefit from support and specialist services, particularly older men and trauma survivors. However, such services were extremely limited in northern Uganda at the time of the study (and indeed elsewhere in Uganda (Tumwesigye & Kasirye, 2005)). The Ugandan government has now introduced a national alcohol policy (albeit a rather contentious one (Bakke & Endal, 2010)). Guidelines on assessing alcohol and other substance use in conflict-affected and displaced populations have also been produced (UNHCR & WHO, 2008). This suggests there may be increasing recognition of the problem of alcohol disorder by the government in Uganda, and also amongst humanitarian agencies.

### 4.1 Study limitations

The validity of the AUDIT instrument and types of reliability such as test–retest reliability were not assessed. However, internal reliability was good (Cronbach's alpha 0.91). The study did not explore family history of alcohol disorder, and there are clearly genetic and parental behavioural influences on alcohol disorder (WHO, 2004). The study was also limited to adults and evidence suggest that alcohol consumption amongst children may be a problem in Uganda (Tumwesigye & Kasirye, 2005). Causation of alcohol disorder also cannot be directly attributed as a cross-sectional design was used in the study. Similarly, the temporal relationship between displacement and alcohol disorder could not be measured using the study design.

## 5 Conclusions

This study provides new evidence on levels and determinants of alcohol disorder amongst forcibly displaced persons in low-income settings. Understanding these determinants can help identify potentially vulnerable groups and target responses more effectively.

## Abbreviations

AUDIT: Alcohol Use Disorders Identification Test
CI: confidence interval
IDP: internally displaced person
OR: odds ratio

## Role of funding sources

This work was supported by the Wellcome Trust [073109/Z/03/Z]. The Wellcome Trust had no role in the study design, collection, analysis or interpretation of the data, writing the manuscript, or the decision to submit the paper for publication.

## Contributors

BR led the study concept and design, data collection, data analysis, and drafting of the manuscript. KFO participated in developing the study concept and design, data collection, review of data analysis, and review of the manuscript. JB participated in developing the study concept and design, review of data analysis, and review of the manuscript. TO participated in developing the study concept and design, data collection, and review of the manuscript. ES participated in developing the study concept and design, and reviewing the manuscript. All authors read and approved the final manuscript.

## Conflict of interest

All other authors declare that they have no conflicts of interest.

## Figures and Tables

**Table 1:** Frequency, typology and categorisation of drinking alcohol, by gender (N = 1206).

	Men		Women		Total	
	N	(%)	N	(%)	N	(%)
*Frequency*						
Never	277	(57.5)	617	(85.2)	894	(74.1)
Monthly or less	29	(6.0)	42	(5.8)	71	(5.9)
2 to 4 times a month	60	(12.5)	34	(4.7)	94	(7.8)
2 to 3 times a week	70	(14.5)	21	(2.9)	91	(7.6)
3 or more times a week	46	(9.5)	10	(1.4)	56	(4.6)

*Typology*						
Abstainer	277	(57.5)	617	(85.2)	894	(74.1)
Infrequent light	80	(16.6)	75	(10.4)	155	(12.9)
Infrequent heavy	9	(1.9)	1	(0.1)	10	(0.8)
Frequent light	108	(22.4)	31	(4.3)	139	(11.5)
Frequent heavy	8	(1.7)	0	(0.0)	8	(0.7)

*Categorisation*						
No alcohol problem	326	(67.6)	673	(93.0)	999	(82.8)
Advice suggested	76	(15.8)	37	(5.1)	113	(9.4)
Counselling suggested	37	(7.7)	4	(0.6)	41	(3.4)
Treatment suggested	43	(8.9)	10	(1.4)	53	(4.4)

Notes: Current abstainer: Never had a drink or had none in the past year. Infrequent light drinker: drinking less than weekly, always < 5 drinks per occasion. Frequent light drinker: drinking weekly and < 5 drinks per occasion. Infrequent heavy drinker: drinking less than weekly, sometimes ≥ 5 drinks per occasion. Frequent heavy drinker: drinking weekly and ≥ 5 drinks per occasion. No alcohol problem (AUDIT score ≤ 7); advice suggested (AUDIT score 8 to 15); Counselling suggested (AUDIT score 16 to 19); Treatment suggested (AUDIT score ≥ 20).

**Table 2:** Characteristics associated with alcohol disorder (N = 1201).

Characteristic	Bivariate analysis			Multivariate analysis		
	OR	[95% CI]	P	OR	[95% CI]	P
Gender:						
Women	Ref			Ref		
Men	**6.26**	**[4.44; 8.81]**	**0.00**	**7.21**	**[4.79; 10.86]**	**0.00**
Age:						
18–29 years	Ref			Ref		
30–39 years	**2.32**	**[1.53; 3.52]**	**0.00**	**2.32**	**[1.57; 3.44]**	**0.00**
40–49 years	**2.66**	**[1.67; 4.22]**	**0.00**	**2.94**	**[1.74; 4.98]**	**0.00**
50 years and over	**3.89**	**[2.47; 6.11]**	**0.00**	**4.14**	**[2.62; 6.52]**	**0.00**
Trauma variables^a^:						
Imprisonment	**2.19**	**[1.59; 3.01]**	**0.00**	1.18	[0.76; 1.84]	0.45
Brainwashing	**1.36**	**[1.00; 1.83]**	**0.05**	1.04	[0.67; 1.60]	0.86
Isolation	**1.88**	**[1.39; 2.55]**	**0.00**	1.35	[0.86; 2.15]	0.19
Escaped death	**1.63**	**[1.14; 2.32]**	**0.01**	1.13	[0.77; 1.66]	0.52
Separation	**1.71**	**[1.26; 2.31]**	**0.00**	0.95	[0.55; 1.65]	0.86
Abducted	**1.68**	**[1.24; 2.27]**	**0.00**	0.74	[0.46; 1.18]	0.20
Beaten or tortured	**1.97**	**[1.43; 2.72]**	**0.00**	1.36	[0.83; 2.24]	0.22
Cumulative trauma exposure^b^:						
0–3 events	Ref			Ref		
4–7 events	1.81	[0.86; 3.77]	0.12	**1.98**	**[1.01; 3.88]**	**0.05**
8–11 events	**2.22**	**[1.07; 4.60]**	**0.03**	**2.00**	**[1.01; 3.97]**	**0.05**
12–16 events	**3.12**	**[1.49; 6.53]**	**0.00**	**2.11**	**[1.02; 4.38]**	**0.04**

Abbreviations: CI, confidence interval; OR, odds ratio. Data highlighted in bold are statistically significant at P < 0.05.

a Reference categories are not experiencing the trauma event.

b Cumulative trauma exposure analysed separately from individual trauma exposure variables in the multivariate analysis.
